# Discontinuation of benralizumab in Canadian patients with severe eosinophilic asthma

**DOI:** 10.1183/23120541.00465-2021

**Published:** 2021-12-06

**Authors:** Stephen G. Noorduyn, Karissa Johnston, Kathy Osenenko, Niroshan Sriskandarajah, Alain Gendron, Lawrence Mbuagbaw

**Affiliations:** 1 AstraZeneca Canada Ltd, Mississauga, ON, Canada; 2 Dept of Health Research Methods, Evidence, and Impact, McMaster University, Hamilton, ON, Canada; 3 Broadstreet HEOR, Vancouver, BC, Canada; 4 Memorial University, St John's, NL, Canada; 5 Dept of Medicine, University of Montreal, Montreal, QC, Canada

## Abstract

Nearly 3.8 million Canadians live with asthma and ∼8% of these patients have severe disease, suffering from frequent exacerbations and leading to increased healthcare costs [1, 2]. In Canada, adult patients with severe eosinophilic asthma who are inadequately controlled on standard therapy may be eligible for treatment with a biologic [3]. Biologic treatments target inflammatory pathways involved in asthma pathogenesis, predominantly endotypes with type 2 inflammation [3, 4].

## Abstract 2

**Within 1 year, 15% of Canadian patients with severe asthma discontinue benralizumab. The reasons for discontinuation are not yet characterised.** https://bit.ly/3kaRDlp

*To the Editor*:

Nearly 3.8 million Canadians live with asthma and ∼8% of these patients have severe disease, suffering from frequent exacerbations and leading to increased healthcare costs [1, 2]. In Canada, adult patients with severe eosinophilic asthma who are inadequately controlled on standard therapy may be eligible for treatment with a biologic [3]. Biologic treatments target inflammatory pathways involved in asthma pathogenesis, predominantly endotypes with type 2 inflammation [3, 4].

Benralizumab was approved in Canada in February 2018 as an add-on maintenance therapy for patients with severe asthma [5]. Access is predominately restricted to those who meet specific clinical criteria and benralizumab is distributed through a patient support programme (PSP) that serves most patients (≥95%) receiving therapy in Canada. The administrative data from this programme can be used to evaluate persistence on therapy for those initiating therapy with benralizumab.

This paper represents an early phase from the Canadian contribution to the XALOC multicountry real-world evidence programme. This programme encompasses several areas of research that will provide additional clinical insight beyond the findings detailed here.

Within this retrolective study, we detail real-world discontinuation of benralizumab using administrative data from the Canadian PSP. All patients who received at least one injection of benralizumab were eligible for inclusion in the analysis. Patients were followed until either treatment discontinuation or the end of follow-up period (September 2019), at which point they were censored. Ethics approval was granted by Advarra Institutional Review Board and patients provided informed consent prior to data collection.

The primary outcome was time to treatment discontinuation, measured as the difference between the date of first administration and the date of final injection. Discontinuation was defined as the date of last injection if no further appointments were scheduled. Patients with scheduled appointments were censored at the end of follow up.

We captured age (years), sex, province of origin and self-reported biologic treatment history (mepolizumab, omalizumab or reslizumab). Categorical data was summarised as n (%) and continuous data were summarised as mean±sd. Kaplan–Meier analysis was conducted to describe time from benralizumab initiation to treatment discontinuation, both overall and stratified by prior biologic treatment history. Cox regression analysis was conducted to explore the impact of age, sex, province and treatment history with biologics.

As of September 2019, administrative data were available for 2186 adult patients who had received at least one injection benralizumab from one of 136 clinics across Canada between April 2018 and May 2019. The mean age was 56.7±14.1 years and the majority (57.8%) were female. Median (interquartile range) follow-up time was 7.6 (3.7–11.2) months. Patients were included from all Canadian provinces and territories with the following distribution: 64.9% in Central Canada, 27.5% in Western Canada and the Prairies, 7.4% in Eastern Canada, and 0.2% in Territories and Nunavut.

Of 2186 total patients included in this analysis, 8% (95% CI 7.2–9.7%) and 15% (cumulative, 13.2–17.4%) discontinued therapy at 6 months and 12 months post-initiation, respectively. Among those who discontinued treatment, the mean time to discontinuation was 4.6±3.7 months with a mean of 4.2±2.1 injections prior to discontinuation. The probability of discontinuation was statistically indistinguishable when stratified by prior biologic exposure (n=216) (figure 1a). Of the 228 patients who discontinued, 65.4% were female, 8.4% higher than the proportion on therapy at the end of follow-up (57.0% of 1958 patients). No differences in discontinuation rates were noted by age, province or treatment history.

**FIGURE 1:**
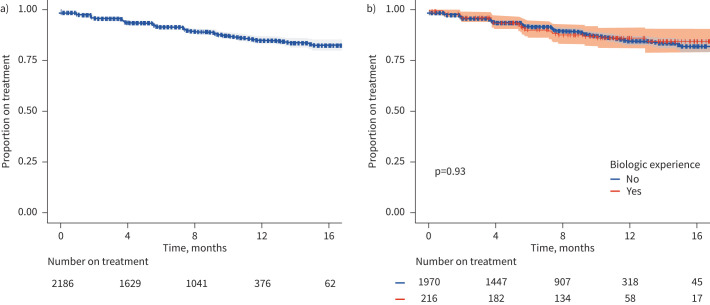
Time to treatment discontinuation for patients initiating treatment with benralizumab in Canada a) overall and b) stratified by prior biologic exposure.

To our knowledge, this is the first report on real-world use of benralizumab in Canada and represents an important early finding from clinical practice for treatment of patients with severe eosinophilic asthma. Given the cost of biologics for treatment of severe asthma, special authorisation is required for access to and reimbursement of these therapies in Canada. As a result, discontinuation rates within this large cohort are a relevant outcome for physicians and payers, who may see this as a useful surrogate for clinical outcomes or funding decisions (*e.g.* patient response or drug wastage).

Interestingly, this analysis showed no difference in the discontinuation rates of patients who had been previously treated with another biologic relative to naïve peers, indicating benralizumab may act similarly in patients with or without previous biologic treatment history. While this may provide early evidence to address the question of therapeutic sequencing, there remain unanswered questions in this area, including analyses which control for known confounders such as the disease phenotype (*e.g.* eosinophilic or IgE driven, or both) and severity.

There are some limitations to this study, most related to the nature of the data utilised. Although the patients included represent a near-complete population estimate, the analysis is based on administrative data collected to support injection and treatment scheduling. As a result, the reasons for discontinuation cannot be determined within these data and may range from administrative reasons to safety concerns. In addition, available patient characteristics are limited to basic demographic information and treatment histories are self-reported, collected only as necessary to support PSP delivery. Therefore, this analysis represents a snapshot of treatment patterns of benralizumab in Canada, similar in scope and limitations to other recent studies [6, 7].

A number of real-world studies has reported discontinuation rates of other biologics for severe asthma across Europe and Canada. In 2020, an observational study of patients receiving omalizumab in Canada reported that 29.5% (n=342) of patients discontinued treatment at 12 months with an additional 17.8% (n=206) and 23.5% (n=272) discontinuing treatment by 24 months and 36 months [6]. Another Canadian study with similar methods and secondary data sources assessed the persistence of mepolizumab therapy and reported 40.3% of 1441 patients had discontinued at 12 months, increasing to 57% (95% CI 54–61%). It must also be noted that these patients were considered discontinued if they remained untreated for at least 90 days (three consecutive missed treatments) after their final treatment [6, 7]. While our definition is potentially more sensitive to early drop-out, the time to discontinuation reported here is lower than observed for other biologic therapies in a similar patient population [6, 7]. However, these are separate observational studies and care should be taken to avoid cross-study comparisons.

While some clinical features (IgE levels, eosinophil count, patient age and dosing/mode of delivery considerations) may impact the relative suitability across biologic agents, the choice of optimal biologic therapy is not always clear. Benralizumab has a distinct mechanism of action with a long dosing period, and this could lead to differences in patient preference, clinical outcomes and disease profile. Nonetheless, further detail encompassing the patient characteristics, performance while on therapy, rationale for discontinuation and outcomes following discontinuation is needed to better understand factors associated with treatment success.

In summary, 15% of severe asthma patients that initiated therapy with benralizumab discontinued their treatment by the end of the first year of therapy. Other available biologic therapies in severe asthma have demonstrated a substantially higher rate of discontinuation in different datasets in Canada. The clinical and patient factors associated with discontinuation of therapy (and the corollary, persistence on therapy) remain relatively uncharacterised in this patient population. Further study is ongoing within the XALOC multicountry research programme to further understand the clinical rational for persistence on therapy, including treatment response and patient preference.
